# Antimicrobial functional ingredients used in hand hygiene products: a cross-sectional survey on regional e-commerce platforms in Wuhan area of China

**DOI:** 10.2478/abm-2025-0037

**Published:** 2025-12-31

**Authors:** Xintong Chen, Jun Wang

**Affiliations:** Institute of Pharmaceutical Innovation, Department of Pharmacy, Hubei Province Key Laboratory of Occupational Hazard Identification and Control, School of Medicine, Wuhan, University of Science and Technology, Wuhan 430065, China; Institute of Pharmaceutical Innovation, Department of Pharmacy, Hubei Province Key Laboratory of Occupational Hazard Identification and Control, School of Medicine, Wuhan University of Science and Technology, Wuhan 430065, China

**Keywords:** alcohol, antimicrobial functional ingredients, hand sanitizers, post-pandemic era, product survey

## Abstract

**Background:**

Hand hygiene products containing antimicrobial functional ingredients have become daily products for many people during the post-pandemic period. However, their current market scenarios have not been well explored.

**Objective:**

To provide an overview of antimicrobial functional ingredients in hand sanitizer products marketed to the public in Wuhan, China.

**Methods:**

This cross-sectional product survey was conducted in September 2023. Information about existing hand hygiene products available online for household use was obtained from 2 major local e-commerce platforms in the Wuhan area of China: Tmall Supermarket and Jingdong Supermarket.

**Results:**

A total of 674 hand sanitizer products were included in the survey, which were evenly divided into alcohol-based and alcohol-free sanitizers. More than 22 non-alcoholic compounds were used in alcohol-free hand sanitizer products, among which the top 5 major ingredients were benzalkonium chloride (BAC), iodine-based compounds, chloroxylenol (CX), hypochlorous acid, and polyhexamethylene biguanide. A majority (83.2%) of hand sanitizer products available on e-commerce platforms were single-ingredient formulations, with the remaining 16.8% containing sanitizers in combination. Triclosan (TCS)/triclocarban (TCC)-containing products accounted for 4.2% of all the included products and 8.3% of alcohol-free products; their recognized alternatives BAC/benzethonium chloride/CX had replaced the role of TCS/TCC in hand sanitization, especially in liquid hand rubs, antiseptic hand wipes, and liquid soaps. A total of 13 natural antimicrobial ingredients were identified in 2.1% of all the products.

**Conclusion:**

There was a booming and diversified hand sanitizer market in Wuhan, China during the post-pandemic era. A market shift from alcohol-based to alcohol-free hand sanitizers has emerged.

Based on the crucial role of hand-to-mouth and other hand-to-face contacts in the transmission of pathogenic microorganisms, including severe acute respiratory syndrome coronavirus 2 (SARS-CoV-2) virus, hand hygiene through the use of hand sanitizers is officially encouraged by governments and public health agencies around the world to prevent infections and epidemic diseases such as the coronavirus disease 2019 (COVID-19), especially when the water and soap are not readily available for handwashing [1]. Considerable consumers have the perception that hand sanitizers are more effective in killing the SARS-CoV-2 virus than handwashing with water and ordinary soap [1, 2]. Following the enhanced self-protection awareness triggered by COVID-19, accumulative evidence [1–3] has shown that the demand for hand hygiene products among the public has shifted toward those containing antimicrobial functional ingredients such as alcohol and other biocidal agents, resulting in an upward trend of global hand sanitizer market during the post-pandemic period. In fact, hand sanitizers have already become one of the daily products for many people after COVID-19 pandemic [1, 2, 4]. During various periods following the onset of COVID-19, the proportion of consumers using hand sanitizers has been reported to be approximately 89.8% in Korea [1], 77.9% in Iran [5], and 99.6% in China [2], respectively. Moreover, along with pandemic-related changes in public hygiene habits [1, 2], it can be speculated that hand sanitizers would be increasingly consumed in household settings during the post-pandemic era.

In order to meet the diversified needs of various consumer groups, there are a variety of commercially available hand sanitizer products containing numerous antimicrobial functional ingredients; however, their efficacy and safety differently vary [6]. Based on their rapid and broad-spectrum microbicidal activity, alcohol-based hand sanitizers with a concentration of 60%–95% ethanol or isopropanol have been strongly recommended by the World Health Organization (WHO) [7] for preventing the transmission of coronavirus. However, if alcohol-based hand sanitizer products are improperly stored or applied, their flammability might pose fire hazards and burn injuries for users. Moreover, certain adverse health effects such as skin and eye injury, methanol poisoning, and even death, have been closely associated with accidental or intentional exposures to alcohol-based hand sanitizers [5, 7]. Especially under the circumstance of reduced global alcohol supply chain resulting from the surge in market sales of alcohol-based hand sanitizers after COVID-19 outbreak, alcohol-free products have been widely available on the market [8]. Nevertheless, unlike the alcohol-based formulations, no standardized formula following officially recommended ingredients and their concentrations exists for alcohol-free hand sanitizers. Currently, the antimicrobial functional ingredients in alcohol-free hand sanitizers are extremely diverse in terms of chemical structure, antimicrobial mechanism, and biological activity [9, 10]. In fact, because of the general lack of necessary training from official and reputable authorities, the vast majority of mass consumers have little familiarity with disinfectants in hand sanitizers, thereby resulting in irrational consumption behavior, ineffective hand disinfection, even poisoning, and other health problems in users [2, 5]. Therefore, it is necessary to perform a survey of antimicrobial functional ingredients in hand sanitizers marketed to the public available for purchase, especially during the post-pandemic era.

As a well-developed first-tier metropolitan city with over 13 million people located in central China, Wuhan is the original epicenter of COVID-19, where its outbreak was first reported in late December 2019. Under unprecedented stressors of the COVID-19 pandemic, Wuhan citizens have to improve their individual compliance with preventive health behavior [11]. Currently, online e-commerce platforms have been an important sales channel for hand hygiene products for household use in China. This study aimed to provide an overview of antimicrobial functional ingredients in hand sanitizer products marketed to the public available for purchase on regional e-commerce platforms in Wuhan, China, during the post-pandemic period.

## Methods

This cross-sectional product survey was conducted in September 2023. Information about existing proprietary hand hygiene products available online for household use was obtained from 2 leading local online markets within the Wuhan area: Jingdong Supermarket and Tmall Supermarket. We chose Jingdong Supermarket and Tmall Supermarket as the sources of data because they are the first and second largest local e-commerce platforms in China, respectively, thus ensuring adequate reflection of consumer behavior in the area. Due to their market dominance, extensive product offerings, and strong presence in the Wuhan area, these platforms might be ideal for capturing a comprehensive snapshot of the local hand sanitizer market. Moreover, good user interfaces of these 2 platforms that are easy to access and extract detailed product information are crucial for an accurate survey.

All available hand sanitizer delivery systems, such as hand rubs (in foam, gel, and liquid), antiseptic hand wipes, and antimicrobial hand soaps in the solid or liquid state [12] were included. We put “hand sanitizer,” “hand rub,” “antiseptic hand wipe,” and “antimicrobial hand soap” as keywords, and a total of 3,270 pieces of information about hand hygiene products were obtained. Subsequently, the obtained information was manually screened. Hand sanitizer products without accurately labeled antimicrobial ingredient information were excluded. The functional ingredients labeled on each product were compared with the disinfectant list in the Guideline for the Use of Disinfectants officially issued by the General Office of the National Health Commission of the People’s Republic of China [13] to determine whether the product was accurately labeled. Finally, 674 products were available for analysis of data on the antimicrobial functional ingredients used in hand hygiene.

The collected data were recorded in Excel^®^ spreadsheets and analyzed using SPSS26.0 (IBM Corp, Armonk, New York). Descriptive data were shown as number (percentage). Comparisons of hand hygiene products containing triclosan (TCS)/triclocarban (TCC), their popular chemical alternatives benzalkonium chloride (BAC), benzethonium chloride (BEC), chloroxylenol (CX), and natural antimicrobial ingredients were performed using multiple chi-square tests or Fisher’s exact tests. *P* < 0.05 or 0.01 were deemed statistically significant.

## Results

**Figure 1.**
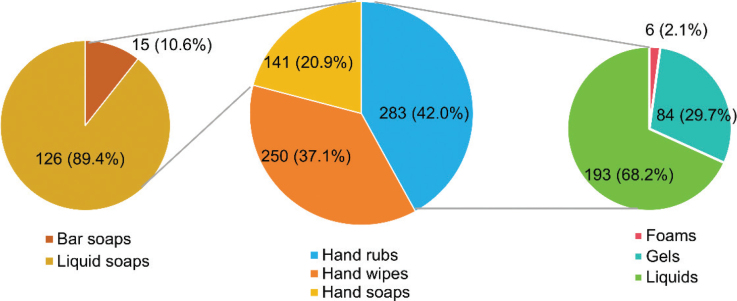
Distribution of sanitizer delivery systems for hand sanitizer products included in this study (n = 674)

As shown in Figure 1, among a total of 674 hand sanitizer products that were collected, waterless hand rubs were the most common hand sanitizer delivery system, accounting for nearly half (42.0%) of all the included products. Thereinto, liquid hand rubs were more available than their gel and foam counterparts on local e-commerce platforms in Wuhan. Moreover, 250 (37.1%) were antiseptic hand wipes, which also acted as an important sanitizer delivery system for hand hygiene products. Among the on-market antimicrobial hand soaps, liquid soaps were more popular than bar soaps.

**Figure 2.**
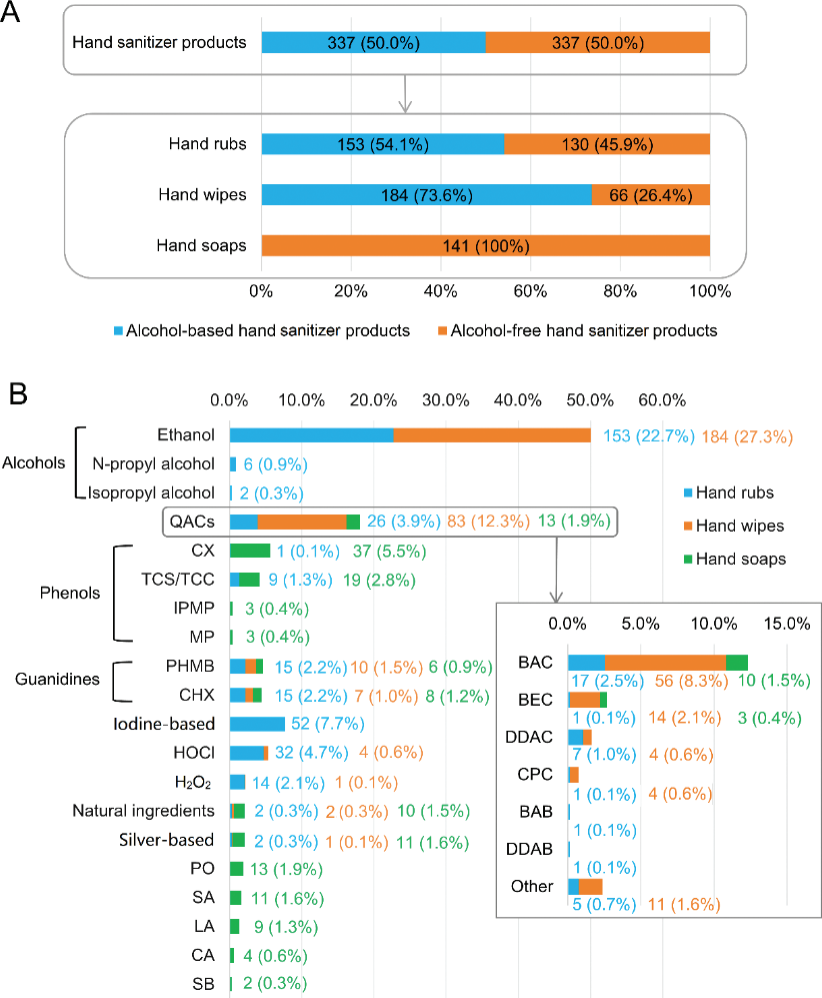
Distribution of different antibacterial functional ingredients in hand sanitizer products included in this study (n = 674). (A) Proportion of alcohol-based and alcohol-free hand sanitizer products. (B) Prevalence of functional ingredients in hand functional products.

The distribution characteristics of the different antibacterial functional ingredients in the 674 hand sanitizer products are shown in Figure 2. All included products were evenly split between alcohol-based and alcohol-free products. Of the 337 alcohol-based products, 153 (45.4%) were hand rubs and the remaining (54.5%) were hand wipes (Figure 2A). Ethanol was the dominant alcohol used for hand sanitization. Among the 337 alcohol-free hand sanitizer products, >22 non-alcoholic compounds were used as sanitizers. Among them, 5 major non-alcohol antibacterial ingredients were found to be BAC, iodine-based compounds, CX, hypochlorous acid (HOCl), and polyhexamethylene biguanide (PHMB), which were contained in 24.6%, 15.4%, 11.3%, 10.7%, and 9.2% of all the alcohol-free products, respectively (Figure 2B).

**Figure 3.**
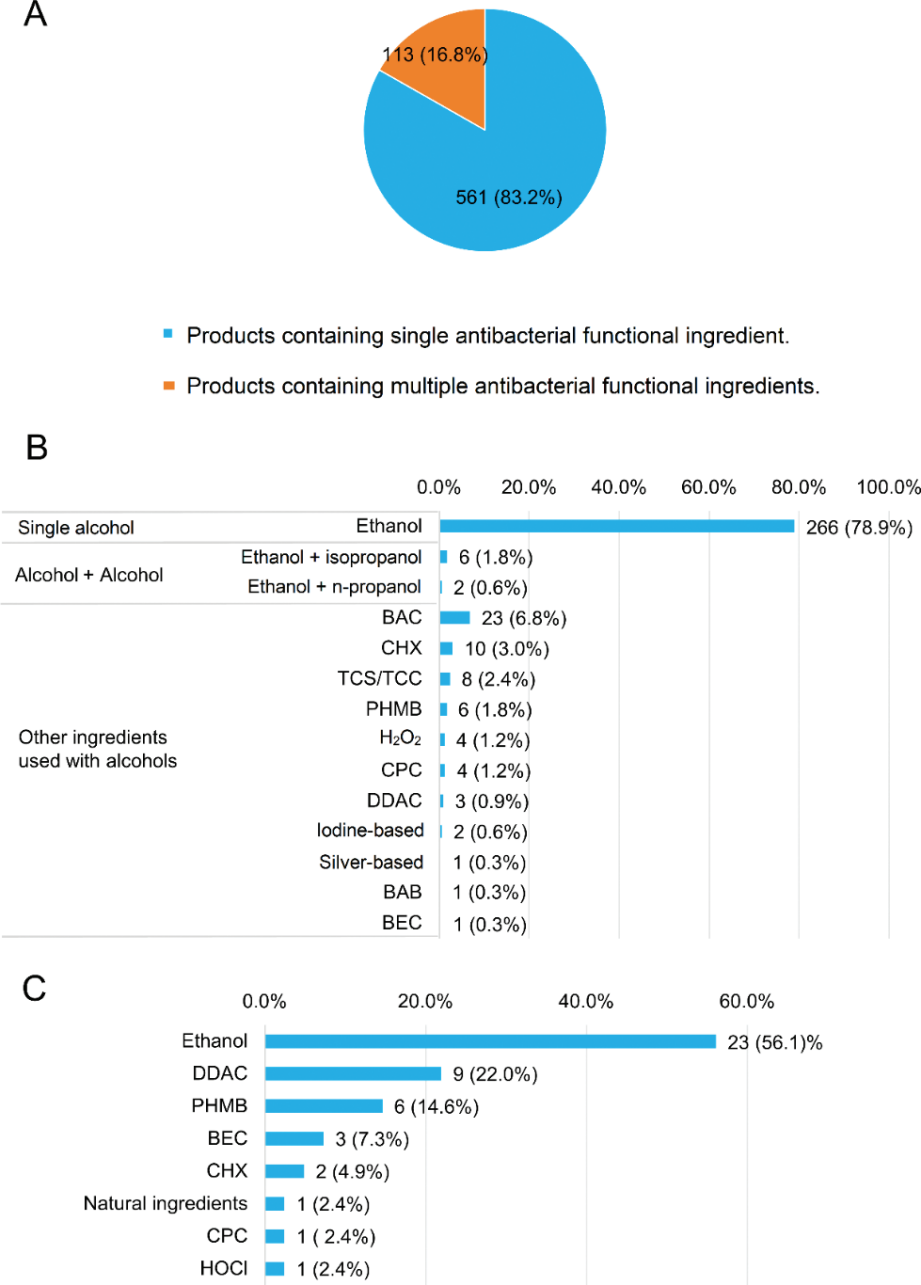
Combination of antibacterial functional ingredients in hand sanitizer products included in this study. (A) Proportion of products containing single and multiple antibacterial functional ingredients (n = 674). (B) Combination of antibacterial functional ingredients in alcohol-based hand sanitizer products (n = 337). (C) Combination of antibacterial functional ingredients in products containing BAC (n = 41). BAB, benzalkonium bromide; BAC, benzalkonium chloride; BEC, benzethonium chloride; CHX, chlorhexidine; CPC, cetylpyridinium chloride; CX, chloroxylenol; DDAC, didecyl dimethyl ammonium chloride; H_2_O_2_, hydrogen peroxide; HOCl, hypochlorous acid; LA, lactic acid; PHMB, polyhexamethylene biguanide; SA, salicylic acid; TCS/TCC, triclosan/triclocarban.

We then explored the combination of antibacterial functional ingredients in the hand sanitizer products included in this survey. As shown in Figure 3A, the majority (83.2%) of hand sanitizer products available on Wuhan’s e-commerce platforms were single-ingredient formulations, with the remaining 16.8% containing sanitizers in combination. Ethanol was the only alcohol used among the 266 single-ingredient alcohol-based products. Among the 71 alcohol-based products containing multiple functional ingredients, 8 products consisted of 2 alcohols, while BAC was the most common non-alcohol sanitizer in combination with alcohols (Figure 3B). The combination of antibacterial functional ingredients in products containing BAC, the latter of which was found to be the most popular non-alcohol hand sanitizer, was further analyzed. In addition to ethanol, as shown in Figure 3C, didecyl dimethyl ammonium chloride (DDAC) and PHMB were relatively frequently used in combination with BAC in on-market hand sanitizer products.

**Figure 4.**
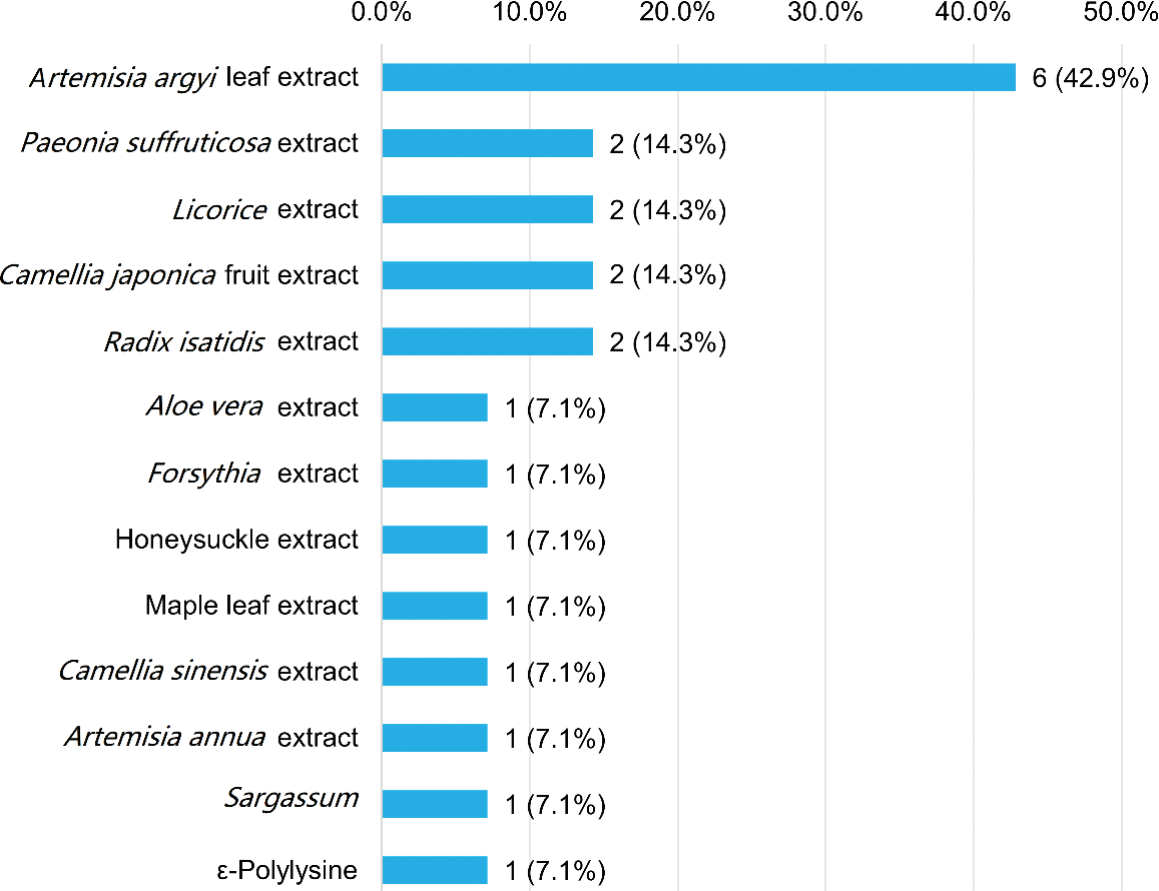
Natural antibacterial functional ingredients in hand sanitizer products included in this study (n = 14).

**Figure 5.**
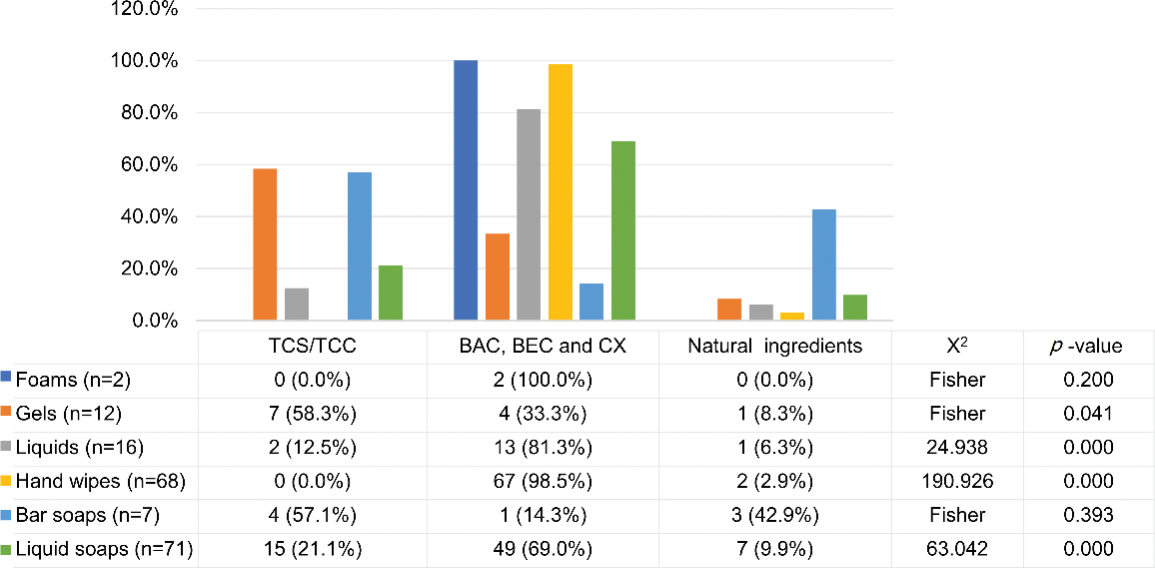
Comparison among hand sanitizer products containing TCS/TCC, their recognized chemical substitutes BAC, BEC, and CX, as well as natural ingredients (n = 176). BAC, benzalkonium chloride; BEC, benzethonium chloride; CX, chloroxylenol; TCS/TCC, triclosan/triclocarban.

A total of 14 hand sanitizer products (2 hand rubs, 2 hand wipes, and 10 soaps) containing 13 natural antimicrobial ingredients, among which extracts from *Artemisia argyi* were added to 6 products, and were thus the most commonly used natural hand sanitizers (Figure 4). We then compared the distribution of products containing TCS/TCC, their recognized chemical alternatives BAC/BEC/CX, and natural antimicrobial ingredients among the different sanitizer delivery systems (Figure 5). Of the 176 products containing these 3 classes of sanitizers, TCS/TCC was found in 15.9% (n = 28), the vast majority (77.3%, n = 136) contained BAC/BEC/CX, and only 8.0% (n = 14) used the natural products as antimicrobial ingredients. Concerning the sanitizer delivery systems, TCS/TCC was significantly more frequently used in hand gels (*P* < 0.05), whereas BAC/BEC/CX was significantly more common in liquid hand rubs, hand wipes, and antimicrobial hand soaps in the liquid state (*P* < 0.01).

## Discussion

Among the 4P elements (product, price, place, and promotion) of traditional marketing mix that has a significant influence on the consumers’ response and ultimately determines the degree of marketing success, the product plays a critical role in customers’ consumption of health-related products, and the product characteristics often determine whether the public will purchase or not [14, 15]. As for hand sanitizer products, Wongso et al. [16] have reported that, as the first dimension of marketing mix, the product showed a significant positive correlation with the potential consumers’ purchasing decisions on hand sanitizer products. Antimicrobial functional ingredients directly determine the disinfection efficacy of hand sanitizer products [17], thus acting as a key product characteristic. In this cross-sectional product survey conducted during the post-pandemic period, we found that there were a total of 674 hand hygiene products containing antimicrobial functional ingredients available for the general public to online purchase, suggesting a booming and diversified hand sanitizer market in the Wuhan area, China. According to current national legislation regarding labels and instructions of disinfection products [18], the on-market disinfection products should be accurately labeled in terms of product name, specifications and dosage forms, main functional ingredients and their contents, types of microorganisms to inhibit or kill, manufacturer name and license number, scope and method of use, precautions, executing standard, production date, and shelf life. However, the label contents of hand sanitizer products were not specifically required in this management standard, which might lead to the missing labels of some key product information, such as functional ingredients, because manufacturers labeled their hand sanitizer products according to the standard of common hygiene products. Moreover, some hand hygiene products might be exaggeratedly advertised as having disinfection properties for commercial purposes. In fact, hand sanitizer products labeled with false, misleading, or unproven claims have been found in markets of many countries [19–22]. For example, although the U.S. Food and Drug Administration regulates alcohol-based hand sanitizers as over the counter (OTC) drugs, a previous product survey found that over 70% of available samples showed a lack of essential labeling information [22]. In the present study, only approximately 20% of so-called hand sanitizer products were eventually included, which indicated somehow the problem of current regulatory mechanism on health-related marketing products. Undefined and nonuniform product types of hand sanitizers might impede the management of on-market hand sanitizer products. With the growing market for hand sanitizers, management standards targeting products for hand disinfection might be required.

In terms of modes of delivering the antimicrobial functional ingredients, whether alcohol or other disinfection agents, waterless hand rubs were more popular than wipes and soaps on the Wuhan online market. Gels, foams, and liquids accounted for 29.7%, 2.1%, and 68.2% of all the included hand rubs, respectively. According to the findings of current published research, it is still difficult to definitely conclude which mode of hand sanitizing delivery format is over the other [18]. As for the anti-bacterial efficacy profile, it has been generally considered that the ethanol gel formulations, unless being specially formulated, are less effective than ethanol solution formulations [23, 24]. However, based on their fast absorption, not sticky, soft and moisturized hand feel, low smell, and clean feel, the gels and foams have been found as the more desirable formats of alcohol-based hand rubs for healthcare workers than liquids/sprays, thus it might lead to better hand sanitization compliance [25]. Moreover, on the Wuhan online hand sanitizer product market, the antiseptic wipes accounted for a considerable proportion. The previous study [26] proposed that using hand wipes has the added benefit that mechanical friction associated with wipes would produce greater reductions in pathogen load, relative to rubs, and thus could be considered an acceptable product for healthcare personnel.

This product survey focused on the antimicrobial functional ingredients of hand sanitizer products. We found that alcohols act as key functional ingredients in hand rubs and wipes. In 2019, the global alcohol-based sanitizer market accounted for about 64% of the hand sanitizer market [27]. Matatiele et al. [28] randomly collected 94 hand sanitizer samples commercially available in Johannesburg, South Africa, from March to June 2020, and only 1 sample was explicitly stated to be alcohol-free. A cross-sectional study [29] conducted in May 2021 showed that the most common active ingredient in 160 top-reviewed commercial hand sanitizers collected from 5 U.S. online retailers, including Walmart, Amazon, Target, Walgreens, and CVS, was ethanol (81.9%). A recent study [30] scrutinized the composition of a total of 79 hand sanitizer products online and offline marketed in India between May 2019 and May 2022 and found that the vast majority (98.73%) were alcohol-based hand sanitizers. Collectively, these survey data from other regions or countries [28–30] suggested that household hand sanitizers with alcohol as the main antibacterial functional ingredient previously dominated the commercial market. However, the present survey conducted in the Wuhan area during September 2023 showed that just a little more than half (54.1%) of hand rubs in formats of gel, foam, and liquid, 73.6% of hand sanitizer wipes, and half of all on-market household products for hand sanitization contained the alcohols as active ingredients. In spite of possible differences among sampling areas, this finding suggests that during the post-pandemic period, non-alcoholic antimicrobial ingredients have gained popularity, at least in the development and market launch of new household hand sanitizer products for manufacturers.

We found that alcohol-free formulations accounted for half of all the included household products for hand sanitization, thus having an identical prevalence as alcohol-based products. This finding is not consistent with the traditional opinion that the need for non-alcohol-based hand disinfectants is far from universal [28–30], suggesting a possible transition from alcohol-based hand sanitizers to non-alcohol-based antibacterial ingredients for hand disinfection at the market level. Experimental evidence [8, 31, 32] has supported that alcohol-based and alcohol-free sanitizers are often equally effective against pathogenic microorganisms after a single application; however, after repeated use, the efficacy of alcohol-containing sanitizers is lower than that of certain alcohol-free sanitizers. Considering their persistent efficacy and non-flammability, alcohol-free hand sanitizers have attracted considerable attention, not only from the academic community, but also from manufacturers as a new market opportunity [9, 33]. Our survey showed that a variety of non-alcoholic substances (>22) were used as antibacterial functional ingredients in alcohol-free hand sanitizer products commercially available in Wuhan’s online market, and the combination of these substances was common but variable in multiple-ingredient products. Therefore, it must be considered that there is no gold-standard formulation of alcohol-free hand sanitizers, even more so when considering that available non-alcoholic hand disinfectants are continually increasing and being applied in combination but still quite unknown with respect to their appropriate composition or possible synergies and antagonisms among them.

However, it has been well documented that the increasing use of non-alcoholic sanitizers has been closely associated with long-term public health and ecologic risks [5, 6, 10, 34–40]. In particular, as traditional antimicrobial ingredients used in hand sanitizers and other personal care products (PCPs), TCS, and TCC have been banned for certain applications, such as shower gel products, OTC handwashing, and antibacterial soaps, by the U.S. FDA in 2016–2017 [40], due to their inefficacy and potentially harmful effects on ecosystems and humans, such as endocrine disruption, reproductive disorders, intestinal damage, carcinogenicity, and induction of antimicrobial resistance. Thus, TCS and TCC as hand sanitizers are anticipated to be gradually superseded by more safer antimicrobial chemicals [40]. BAC, BEC, and CX are commonly used as preservatives in pharmaceutical preparations, and therefore are postulated to be safe from a public health perspective. It has been speculated that the additions of BAC/BEC/CX to PCPs as an antibacterial functional ingredient are increasing [38]. Accordingly, BAC, BEC, and CX have been regarded by manufacturers as the most popular alternatives to TCS and TCC in antibacterial PCPs [34–36]. However, accumulative evidence [34–36] showed that these alternatives might be more toxic than TCS and TCC under many circumstances. Sreevidya et al. [34] explored and compared the toxicological effects of BAC/BEC/CX with those of TCS/TCC in nematode *Caenorhabditis elegans* and zebrafish. Results showed that exposure of zebrafish embryos to all 3 compounds at concentration ranges of 0.05–5 mg/L resulted in hatching inhibition and delay, embryonic mortality, malformations, and neurotoxicity, while BAC at lower 100 μg/L to lower mg/L concentrations exerted comparable toxicity with TCS in the worm from molecular to organismal levels [34]. In particular, as the dominant ingredient widely used in alcohol-free hand sanitizers, BAC has been paid special attention as a high-priority disinfectant emerging contaminant, based on its global occurrence in environmental matrices and potential adverse impacts on non-target organisms, including phytotoxicity, genotoxicity, endocrine disruption and reproductive impairment, respiratory toxicity, behavioral effects, and neurotoxicity [35–37]. Currently, the adaptation of alternative functional ingredients of natural compounds, which are not synthesized purely through industrial processes but mainly by natural organisms such as plants, microorganisms, and animals, in hand hygiene products has emerged as a promising solution in response to the health and ecological risks posed by chemical-based hand sanitizers currently in use [10, 38, 41]. Therefore, a comparison of hand hygiene products containing TCS/TCC, their popular alternatives BAC/BEC/CX, as well as natural antimicrobial ingredients was a focus of research.

The results of the current study showed that, in an actual market scenario, the alternatives BAC/BEC/CX had really replaced the role of TCS/TCC as biocides in hand sanitization, especially in liquid hand rubs, antiseptic hand wipes, and antimicrobial hand soaps in liquid state; however, TCS/TCC remained as common non-alcoholic active ingredients in hand gels. On the whole, TCS/TCC-containing products only accounted for 4.2% of Wuhan’s household hand sanitizer market (8.3% of alcohol-free products), which was much lower than the previously reported TCS occurrence of 21.34% (35 out of 164) [4] and 31.7% [39] in hand sanitizers in Chinese markets before the pandemic. These data suggested that, despite the fact that TCS and TCC are still available as active ingredients for hand hygiene in the Chinese market, their roles are gradually replaced by surrogate antibacterial ingredients such as BAC/BEC/CX, perhaps due to the well-known healthy and environmental risks posed by TCS/TCC [40]. As for manufacturing enterprises, it is necessary to take the initiative to find substitutes for TCS/TCC to adapt to regulations to increase exports [39]. Currently, on-market non-alcohol hand sanitizers were found in this study to be dominated by BAC, which was presented in 12.3% of all the included hand sanitizer products. This proportion was close to that reported in the U.S. study [29] conducted in May 2021, which found BAC was contained in 9.4% of the included commercial hand sanitizers.

In a search for antibiotic hand soaps on the American market [42], there were several consumer products containing plant-based antimicrobials, indicating a consumer shift toward “natural” products. Under the circumstance that common hand disinfectants such as TCS, TCC, and their popular alternatives BAC/BEC/CX have been demonstrated to pose environmental risks as emerging contaminants after consumption, natural disinfectants extracted from natural herbs and microorganisms have been accepted as the safer and reliable “environmentally friendly” alternatives for health and the environment [10, 41]. A recent cross-sectional study [41] showed that most Chinese residents were more willing to pay for natural “green” disinfectants than common ones at the same price, which might establish a Chinese consumers’ propensity to natural antimicrobial ingredients used for hand hygiene. Therefore, there may be an innovative, dynamic, and promising market for hand hygiene products based on naturally derived anti-microbial compounds that allow consumers to choose according to their personal hygiene needs, especially in China. However, our product survey showed that, among all the included 674 hand sanitizer products, only 14 (2.1%) contained herbal extracts and other natural ingredients with biocidal activity, suggesting that natural antimicrobial ingredients are not as prevalent as expected for hand hygiene in the Chinese market. Moreover, the natural antibacterial ingredients in the on-market hand hygiene products in the Wuhan area are extremely diverse. Thus far, there is still no generally accepted natural antimicrobial ingredient that can be used for hand hygiene, which might be a barrier to market development.

This study offered a timely and relevant exploration of the hand sanitizer market in the post-pandemic era, particularly focusing on the diversity and prevalence of antimicrobial ingredients available to consumers in Wuhan. Nevertheless, there were limitations in this survey. The main limitation of the present study is the reliance on e-commerce platforms for data collection. While these platforms are undoubtedly significant market players, they might not capture the full diversity of products available to consumers, especially those sold offline. Moreover, focusing on just 2 e-commerce platforms and the lack of sample size calculation might limit the generalizability of the findings. In addition, this study offered only a snapshot of marketing products. Due to the lack of historical data on the studied local e-commerce platforms, the trends of hand sanitizer market could not be presented in this cross-sectional study, which should be further explored using a prospective study design. This study focused on the capture of a comprehensive snapshot of labeled antimicrobial ingredients in on-market products. However, other elements (price, place, and promotion) of traditional marketing mix, and other dimensions of products that are usually important for consumers, such as the content of functional ingredients, size/volume, brand, and package, should be further considered and analyzed.
